# Recovery trajectories over six weeks in patients selected for a high-intensity physiotherapy program after Total knee Arthroplasty: a latent class analysis

**DOI:** 10.1186/s12891-021-04037-7

**Published:** 2021-02-13

**Authors:** K. E. M. Harmelink, R. Dandis, P. J. der Van der Wees PJ, A. V. C. M. Zeegers, M. W. Nijhuis-van der Sanden, J. B. Staal

**Affiliations:** 1 Radboud university medical center, Radboud Institute for Health Sciences, IQ healthcare, Geert Grooteplein Zuid 21, 6525 EZ Nijmegen, the Netherlands; 2 FysioHolland Twente, Geessinkbrink 7, 7544 CW Enschede, the Netherlands; 3 Department for Health Evidence, Section Biostatistics, Radboud university medical center, Radboud Institute for Health Sciences, Geert Grooteplein Zuid 21, 6525 EZ Nijmegen, the Netherlands; 4 Medisch Spectrum Twente (MST), Department of Orthopedic surgery, Koningsplein 1, 7512 KZ Enschede, the Netherlands; 5 HAN University of Applied Sciences, Musculoskeletal Rehabilitation Research Group, Kapittelweg 33, 6525 EJ Nijmegen, the Netherlands

**Keywords:** Total knee Arthroplasty, Recovery, Physiotherapy program, Latent class analysis

## Abstract

**Background:**

Recovery trajectories differ between individual patients and it is hypothesizes that they can be used to predict if an individual patient is likely to recover earlier or later. Primary aim of this study was to determine if it is possible to identify recovery trajectories for physical functioning and pain during the first six weeks in patients after TKA. Secondary aim was to explore the association of these trajectories with one-year outcomes.

**Methods:**

Prospective cohort study of 218 patients with the following measurement time points: preoperative, and at three days, two weeks, six weeks, and one year post-surgery (no missings). Outcome measures were performance-based physical functioning (Timed Up and Go [TUG]), self-reported physical functioning (Knee injury and Osteoarthritis Outcome Score-Activities of Daily Living [KOOS-ADL]), and pain (Visual Analogue Scale [VAS]). Latent Class Analysis was used to distinguish classes based on recovery trajectories over the first six weeks postoperatively. Multivariable regression analyses were used to identify associations between classes and one year outcomes.

**Results:**

TUG showed three classes: “gain group” (*n* = 203), “moderate gain group” (*n* = 8) and “slow gain group” (*n* = 7), KOOS showed two classes: “gain group” (*n* = 86) and “moderate gain group” (*n* = 132), and VAS-pain three classes: “no/very little pain” (*n* = 151), “normal decrease of pain” (*n* = 48) and “sustained pain” (*n* = 19). The” low gain group” scored 3.31 [95% CI 1.52, 5.09] seconds less on the TUG than the “moderate gain group” and the KOOS “gain group” scored 11.97 [95% CI 8.62, 15.33] points better than the “moderate gain group” after one year.

Patients who had an early trajectory of “sustained pain” had less chance to become free of pain at one year than those who reported “no or little pain” (odds ratio 0.11 [95% CI 0.03,0.42].

**Conclusion:**

The findings of this study indicate that different recovery trajectories can be detected. These recovery trajectories can distinguish outcome after one year.

## Introduction

An increasing number of studies focussed on the effectiveness of total knee arthroplasty (TKA) surgery for people with end-stage osteoarthritis. They showed that on the average 80% of the patients is satisfied one year after surgery [1, 2]. However, the vast majority of studies on the effectiveness of TKA including a postoperative physiotherapy program used a pre-post design or randomized controlled design with measurements at six months or at one year [3, 4]. In most of these studies recovery trajectories have not been analysed so far. Recovery trajectories might provide a more valid recovery parameter as it combines multiple end-points over time. Moreover, recovery trajectories might also be more valuable than a single end-point, to predict accurately how an individual patient is likely to recover [5] and to identify abnormal recovery. In addition to that, identifying patients with similar patterns of recovery may provide novel insights into subgroups of patients that may or may not benefit from specific rehabilitation programs.

Latent Class Mixture Models (LCMM) are widely used statistical models in social, behavioural and medical science. They can be used to identify latent subgroups, classes or clusters of individuals based on their common growth trajectories over time [6]. These models can be seen as an extension of growth models given the assumption of homogeneity of growth parameters within a latent subgroup [7]. The existence of distinct latent groups can reflect some yet unknown influencing variables like comorbidity or other unobserved individual characteristics, or rehabilitation related factors like adherence to exercise or characteristics of the rehabilitation program. In this work, we use LCMM models to identify subgroups of patients based on their recovery trajectories after TKA surgery. We did this for the first step in a sample of patients included for a high-intensity physiotherapy program after TKA, because these patients were measured frequently. Moreover, the physiotherapy treatment in these patients is equal for all patients, so it is unlikely that this affects the recovery trajectories identified.

We hypothesized that there are different subgroups of patients with distinctly different recovery trajectories after TKA as reported by others [8, 9]. Recovery trajectories were studied earlier in other populations, such as low back pain, [10] neck pain, [11] stroke patients, [12] knee osteoarthritis [13] and once in patients after TKA [14]. However, the latter study only looked at the recovery trajectories between one and five years, [14] while it is well-established that recovery primarily takes place in the first weeks after TKA, [15–18] and that the one-year outcome corresponds mostly with the end-stage in recovery after TKA [15]. There is currently a paucity of information in the literature on recovery trajectories in patients after TKA during the first weeks after surgery. Therefore, the primary aim of this study was to determine if it is possible to identify recovery trajectories for physical functioning (performance-based and self-reported) and pain over six weeks in patients after TKA.

The secondary aim is to explore associations of these early recovery trajectories over six weeks with physical functioning (performance-based and self-reported) and pain after one year.

## Material and methods

### Study design

**Fig. 1:**
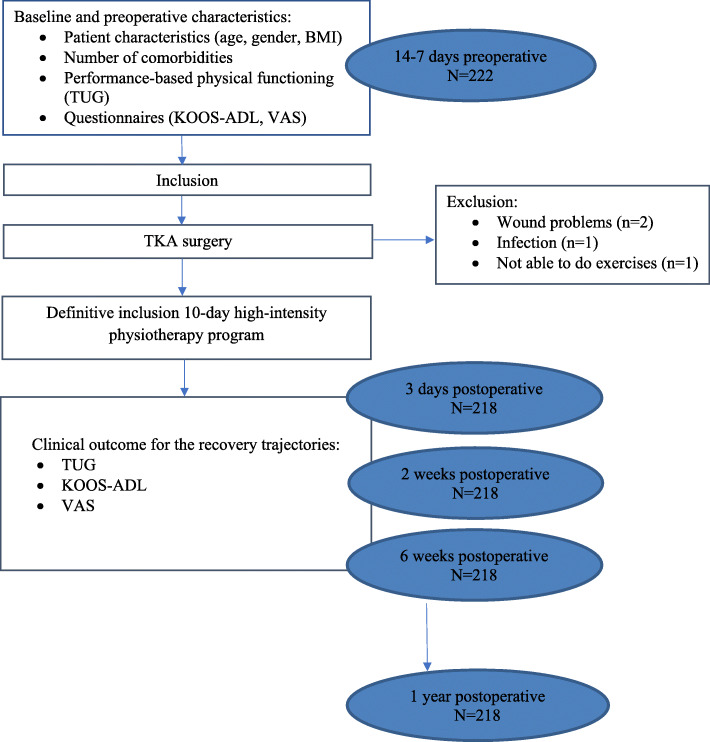
Study flow chart

This is a prospective cohort study with clinical data. The flowchart of this cohort study is presented in Fig. 1. We collected preoperative data between one and two weeks before TKA surgery. Follow-up measurements were performed three days, two weeks, six weeks and one year after surgery. Data collection was performed as part of routine care. We reported this study in accordance with the STROBE statement for the reporting of observational studies [19]. The medical ethical review board of the Medisch Spectrum Twente (MST), Enschede, The Netherlands approved the study (Kh 13–06). All patients provided written informed consent prior to enrolment in the study.

### Participants and setting

Participants were recruited from the MST community hospital in Enschede, the Netherlands between February 2011 and December 2014. Patients who followed a high-intensity physiotherapy program after TKA were included. The decision whether a patient was eligible to participate in the program was made by the orthopaedic surgeon and the physiotherapist together. A requirement was that patients are able to maintain the high-intensity physiotherapy program. Therefore, the following inclusion criteria were mandatory: 1) 18 years or older and diagnosed with primary osteoarthritis; 2) admitted for TKA surgery; 3) preoperatively independent in activities of daily living; 4) no comorbidity that hindered doing exercises [20]; 5) no mental disorders [20] as reported by the patient; 6) physically able and willing to perform a 10-day high-intensity physiotherapy program; and 7) signed informed consent (see Appendix 1). The high-intensity physiotherapy program is explained in Appendix 1.

A proper sample size is important for obtaining adequate statistical power as well as reducing bias related to parameter and standard error estimates for both analyses. An insufficient sample size can be particularly problematic when conducting latent class analyses because it is often associated with convergence issues and perhaps inability to identify small but meaningful subgroups. Unfortunately, determining the sample size needed to conduct a LCMM is not straightforward. “Rules of thumb” (e.g., 5 or 10 observations per estimated parameter) are commonly used to justify a particular sample size. For the multivariate linear regression analysis we apply the rule of thumb that we needed at least 10 patients per variable. A proper sample size for this study is a minimum of 100 patients. Assuming a dropout rate of 15% a total number of 115 patients is appropriate.

### Surgery and physiotherapy

After inclusion in the study by the orthopaedic surgeon and the physiotherapist, the preoperative assessment took place. Thereafter, all participants received a TKA procedure. Five orthopaedic surgeons performed all TKA surgeries following the same surgical procedure. The number of TKA procedures per year carried out by each surgeon varied from 50 to 70. Participants started their rehabilitation during the three day hospital admission. The orthopaedic surgeon and the physiotherapist checked a second time if inclusion in the high-intensity program was possible (no complications which hindered following a 10-day high-intensity program). After discharge and definitive inclusion, participants stayed at a resort where they followed a 10-day high-intensity physiotherapy program, which is explained in Appendix 1. After ten days all participants returned home. The program was available for all patients, independent of their social and economic status. Patients were advised about the continuation of physiotherapy after the high-intensity program, dependent on their physical status [21].

### Measurements

The following variables were collected pre-operatively: patient characteristics (i.e. age, gender, body mass index [BMI], number of comorbidities), performance-based measure (i.e. Timed Up and Go [TUG] [22] and self-reported measure (i.e., physical functioning measured with the Knee Osteoarthritis and Outcome Score Activities of Daily Living scale [KOOS-ADL], [23] and pain measured with the Visual Analogue Scale [VAS]) [24].

Physical functioning and pain are core outcome measures for people undergoing TKA surgery, [25] which was confirmed by patients and orthopaedic surgeons [26]. Mizner et al. advised using both performance-based measure and patient-reported measure for measuring physical functioning [27]. Therefore, performance-based physical functioning (TUG), self-reported physical functioning (KOOS-ADL) and pain (VAS) were used as outcome measures. The recovery trajectories were based on measurements preoperatively, and three days, two weeks and six weeks after surgery. All outcome measures were also measured at one year after surgery.

The TUG is a multi-activity measure [22]. Patients were asked to stand up from an armchair (with a seat height of 46 cm), walk three metres, turn and walk back to the armchair without assistance. For scoring the TUG, the time in seconds to complete the task was measured. The instructions were to walk safely, but as fast as possible. The test was assessed twice and the lowest time score was used as outcome measure.

Self-reported physical functioning was measured with the KOOS. The KOOS is composed of five separately scored subscales: pain, symptoms, ADL, activities in sports and recreation, and knee-related quality of life [23, 28]. Answers were given using a Likert scale, and each question was answered with a score from 0 to 4. A normalised score from 0 to 100 was calculated for each domain (100 indicates no symptoms/pain, and 0 indicates extreme symptoms/pain). The KOOS has excellent reliability and good content and construct validity when used for short- and long-term follow-up of knee injury [29, 30]. It has been validated for people with TKA [29–31]. The score per subscale was determined and only the subscale ADL was used to measure outcome.

The VAS was used to measure pain [24]. Patients were asked to mark on a 100-mm line their pain rating during the last week, where 0 corresponded to no pain and 100 corresponded to worst imaginable pain.

### Statistical analysis

Patient baseline and preoperative characteristics were described as median [interquartile range (IQR)] or number of patients (percentage). The subpopulations of patients based on the postoperative performance-based physical functioning and self-reported physical functioning outcome trajectories and pain outcome trajectories during six weeks were identified using latent class mixed model (LCMM). The LCMM finds potential latent profiles in heterogeneous populations. It combines a latent class model to identify homogenous latent classes of subjects and a mixed model to describe the mean trajectory over time in each latent group, while taking into account the individual correlation between repeated measures. Each subpopulation has its own physical functioning (TUG, KOOS-ADL) or pain (VAS) growth parameters. We fitted the models using not only linear functions of time but also quadratic functions to allow nonlinear mean trajectories over time. The optimal number of classes was determined using a forward procedure, starting with one class and no subpopulations in the study sample. Then one class was added for each model. To evaluate if the model with one added class improved, three steps were taken: 1) The Bayesian information criterion (BIC) was used [10, 13, 32]. The BIC considered the likelihood of the model and the number of parameters in the model. A lower BIC value indicates a better model fit [33] and is a guidance to decide the optimal number of classes [10, 13, 32]; 2) Patients were assigned to their most likely class based on a posterior probability of ≥0.7 [32].

The association of the identified groups with the one year outcomes was analysed using multivariable regression analysis [34]. Because 191 patients scored a VAS 0 (no pain) after one year, we decided to dichotomize the VAS score into ‘no/very little pain’ (VAS score 0–20) and ‘pain’ (VAS score) ≥21. This cut-off point was also used in other studies using the patient acceptable symptoms state (PASS) [35, 36]. Dichotomizing was only done for the one year VAS scores.

The one year responses were regressed on the identified subgroups and on a set of relevant baseline covariates: Age, gender and BMI. Linear regression models were used for the one year TUG and KOOS scores, and a logistic regression model for the one-year dichotomized VAS outcome. The association was determined with the regression coefficient and the 95% confidence interval (linear regression models) for TUG and KOOS and the odds ratio and the 95% confidence interval (logistic regression model) for the VAS of pain during the last week. The overall fit of the models were assessed using the total variance explained, the *R*^2^ for the linear regression models and Nagelkerkes *R*^2^ for the logistic regression model.

Statistical analysis was performed with R software version 3.4.4 [37] and IBM Statistical Package for the Social Sciences (SPSS 25.0), [38] The ‘lcmm’ R package was used to perform the latent class analysis [39].

## Results

### Study population

In total 222 patients were selected and agreed to participate in the 10-day high-intensity physiotherapy program. After surgery four patients were excluded for the 10-day high-intensity training program because of wound problems (*n* = 2), infection (*n* = 1) and not able to do exercises (=1). Therefore, 218 patients were definitively included in the study, as shown in Fig. 1. All measurements at baseline and during follow-up were completed for all included patients. Table 1 shows the baseline characteristics of the study population (*n* = 218).

**Table 1:** Characteristics study population

Characteristic	Total cohort (*n* = 218)
Age (year), median [IQR]	65 [60, 71]
Gender, n (%) female	153 (70.2%)
BMI, median [IQR]	24.81 [22.55, 27.41]
Number of comorbidities, n (%)	
0	59 (27.0%)
1	40 (18.3%)
2	38, (17.4%)
3	37 (17.0%)
4	25 (11.5%)
5	18 (8.3%)
6	1 (0.5%)
Preoperative TUG score (seconds), median [IQR]	8.65 [7.83, 9.88]
Preoperative KOOS-ADL score, median [IQR]	50 [35, 63]
Preoperative VAS score, median [IQR]	52 [42, 62]

BMI = Body Mass Index; IQR = interquartile range; KOOS-ADL = Knee injury and Osteoarthritis Outcome Score-Activities of Daily Living; n = number of patients; TUG = Timed Up and Go; VAS = Visual Analogue Scale

### Recovery trajectories

#### Performance-based recovery trajectories for physical functioning (TUG)

Appendix 2 shows the BIC from the linear and quadratic models of trajectories for TUG and using different numbers of groups. We selected the quadratic three-class model with the lowest BIC amongst the presented models to be the optimal models.

**Fig. 2:**
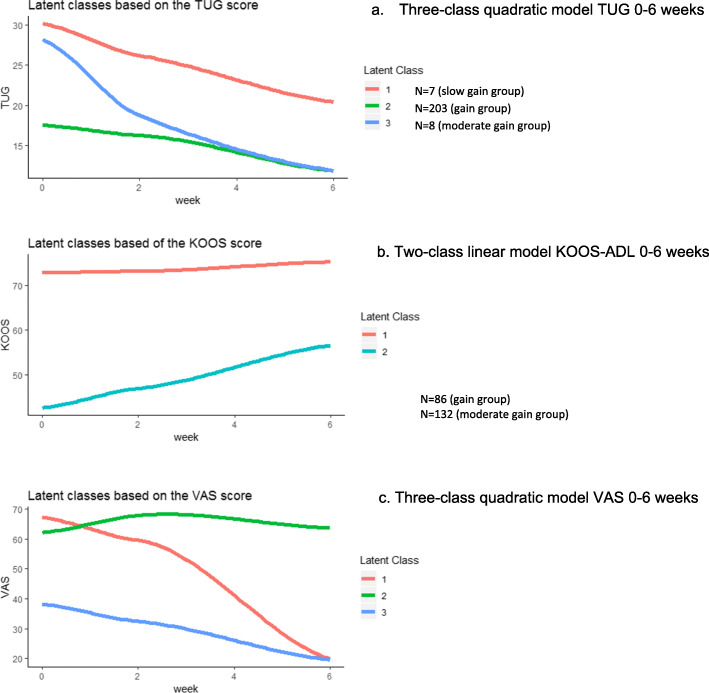
Models for TUG, KOOS-ADL and VAS

Figure 2a shows the mean trajectories per class for the TUG model. The performance-based physical functioning classes were defined as “low gain group” (class 1, *n* = 7), “gain group” (class 2, *n* = 203) and “moderate gain group” (class 3, *n* = 8). In total 211 patients (97%) were recovered (class 2 and 3) and 7 patients (3%) were not recovered (class 1) based on the six weeks recovery trajectories.

#### Self-reported recovery trajectories for physical functioning (KOOS-ADL)

The most optimal LCMM model for KOOS-ADL with the lowest BIC was the linear two-class model (see Table 2). The mean trajectories per class for the KOOS-ADL model are shown in Fig. 2b. The self-reported physical functioning classes were defined as “gain group” (class 1, *n* = 86) and “moderate gain group” (class 2, *n* = 132), so all patients recovered (*n* = 218, 100%).

**Table 2:** The results of multivariable linear and logistic regression models

	One year outcome				
**Multivariable linear regression models**				**Multivariable logistic regression model**	
**TUG**		**KOOS-ADL (range 0–100)**		**VAS-pain (binary)**	
Predictor	*Regression coëfficient [95% CI], p-value*	Predictor	*Regression coëfficient* *[95% CI], p-value*	Predictor	*Odds ratio* *[95% CI], p-value*
Class: “low gain group” versus “moderate gain group”	3.31 [1.52, 5.09], *p* < 0.01	Class: “gain group” versus “moderate gain group”	11.97 [8.62, 15.33], p < 0.01	Class: “normal decrease of pain” versus “no/very little pain”	0.92 [0.17,4.84], *p* = 0.58
Class: “gain group” versus “moderate gain group”	−0.56 [−1.75, 0.64], *p* = 0.36			Class: “sustained pain” versus “no/very little pain”	0.11 [0.03,0.42], *p* = 0.01
Age (year)	0.00 [−0.03, 0.03], *p* = 0.96	Age (year)	0.18 [−0.03, 0.39], *p* = 0.10	Age (year)	0.95 [0.88,1.04], *p* = 0.07
Gender	−0.09 [− 0.58, 0.41], *p* = 0.73	Gender	2.40 [−1.17, 5.98], *p* = 0.19	Gender	0.45 [0.13,1.51], *p* = 0.63
BMI	−0.03 [− 0.10, 0.04],p = 0.36	BMI	0.13 [− 0.38, 0.64], *p* = 0.62	BMI	1.08 [0.9,1.3], *p* = 0.66
Model R^2^	0.14	Model R^2^	0.20	Model R^2^	0.08 ^⊗^

BMI = Body Mass Index; CI = confidence interval; R^2^ = explained variance; *p < 0.05 = statistical significant; ^⊗^ Nagelkerkes R^2^; KOOS-ADL = Knee Osteoarthritis Outcome Score-Activities of Daily Living, TUG = Timed Up and Go; VAS = Visual Analogue Scale

#### Self-reported recovery trajectories for pain (VAS)

The most optimal model retrieved by LCMM was a quadratic three-class model for VAS with the lowest BIC amongst the other presented models (see Table 2). Figure 2c showed the mean trajectories per class for the VAS model. The three classes identified were as follows: “normal decrease of pain” (class 1, *n* = 48), “sustained pain” (class 2 *n* = 19), and “no/very little pain” (class 3, *n* = 151). In total 199 patients (91%) were recovered on pain (class 1 and class 3) and 19 patients (9%) were not recovered (class 2) based upon the six weeks recovery trajectories.

### Association between the identified recovery trajectories and one-year outcomes

#### Performance-based physical functioning

The median TUG score for the total population six weeks after surgery is 11.83 [95%CI 10.41, 12.98] seconds and after one year 8.01 [95% CI 7.15, 8.98] seconds. Table 2 shows the regression coefficients of the multivariable linear regression model for TUG. The” low gain group” (class 1) had a statistically significant lower score than the “moderate gain group” group (class 3) after one year. The “gain group” (class 2) had a non-significant faster score of − 0.56 [95%CI -1.75, 0.64] seconds compared to the “moderate gain group” (class 3) after one year.

#### Self-reported physical functioning

In the total population KOOS-ADL score improved from 65 [95% CI 54, 75] after six weeks to 75 points [95% CI 66, 88] after one year. Table 2 shows the results of the multivariable linear regression model for KOOS-ADL. The “gain group” (class 1) had a statistically significant better score than the “moderate gain group” (class 2) after one year.

#### Pain

Table 2 shows the odds ratios of the multivariable logistic regression model for VAS. After one year 205 patients were categorised as “no/very low pain” (VAS 0–20) and 13 patients were categorised as “pain” (VAS > 20). From the 19 patients with “sustained pain” after six weeks, five patients (26%) experienced sustained pain after one year. In both the “normal decrease of pain” group and the “no/very little pain” group experienced 4% of the patients after one year sustained pain (respectively 2 from the 48 patients and 6 from the 151 patients).

The “normal decrease of pain” group (class 1) had a 8% lower odds to become free of pain after one year than the “no/very little pain” group (class 3), but this was not significant. However, the “sustained pain” group (class 2) had a statistically significant (*p* < 0.001) 89% lower odds to become free of pain after one year than the “no/very little pain” group (class 3).

## Discussion

### Key findings

The purpose of this study was to identify recovery trajectories in patients who attended a high-intensity physiotherapy program after TKA surgery. For performance-based physical functioning three classes were identified: a “gain group” (*n* = 203), a “moderate gain group” (*n* = 8) and a “low gain group” (*n* = 7). Self-reported physical functioning showed two recovery trajectories, namely a “gain group” (*n* = 86) and a “moderate gain group” (*n* = 132). For pain three classes were identified: “no/very little pain” (*n* = 151), “normal decrease of pain” (*n* = 48) and “sustained pain” (*n* = 19). Patients had further improvements for physical functioning and pain between six weeks and one year.

### Strengths and limitations

The longitudinal design with a high follow-up rate is a strength of the study with five measurements over one year. Data were sampled in real clinical practice and patients were tested by the physiotherapist during the high-intensity program and during the regular control visits and comprised of both self-reported and physical performance tests. This minimized the burden of the patients. We expect that different types of physiotherapy treatment programs could have affected the recovery after TKA. However, this was stable in our study, since all included patients followed the same program. Our selection of participants resulted in a relatively homogeneous population. Similar analyses of recovery trajectories need to done in more heterogenous population of post-TKA patients including patients with a less favorable prognosis.

The latent class analysis which is a relatively new method that, helps to utilize all available information in the repeated measurement using flexible random effects models that captures the change of the trajectory over time, while allowing the patients to have different distributions and therefore to be classified to separate homogenous subgroups [32]. LCMM gives an accurate prediction of how an individual patient is likely to recover postoperatively [32] and it identifies abnormal recovery more than simpler approaches. A potential limitation may be the lack of follow-up measurements between six weeks and one year due to practical reasons. The variation in patient profiles is small and room for improvement is limited.

All patients were allowed to recover further with additional physiotherapy sessions after the high-intensity physiotherapy program to optimize their rehabilitation. In another study in a smaller population we found that both patients with a favorable and less favorable recovery after the high-intensity physiotherapy program continued physiotherapy [21]. There was no additive value of prolonged physiotherapy after the high-intensity physiotherapy program in both groups [21]. Therefore, we expect that continuation of physiotherapy after the high-intensity program had no major impact on the further recovery trajectories.

### Clinical implications

Identifying recovery trajectories using LCMM is a relatively new technique, which was as far as known only once researched in patients after TKA between 1 and 5 years after surgery by Dowsey et al. [14] The recovery trajectories we found for pain were not comparable to those found in the study by Dowsey et al. (2015) [14]. In our study 8.7% (*n* = 19) of the patients experienced “sustained pain”, while in the study of Dowsey et al. [14] 21.5% were classified as ‘moderate pain’ (this study identified three classes: “No pain” (33.1%), “Mild pain” (45.4%) and “Moderate pain” (21.5%)) during one till five years postoperative. As shown our population showed better results on pain. This difference could be explained due to the combination of the high-intensity physiotherapy program in a selected group of patients which led to better results. For self-reported physical functioning similar differences were seen, namely 23.8% in the study by Dowsey et al. [14] were classified as ‘high’ self-reported physical function (this study identified three classes: “high physical functioning (23.8%), “moderate physical function” (54.6%) and “low physical functioning”(21.6%)) against 39.4% (“gain group”) in our study.

Patients in this study showed progress on the measurements between six weeks and one year. The minimal clinical important difference for TUG was 2.27 s, [40] for KOOS 20 points [41] and for VAS 22.6 points, [42] therefore this recovery between six weeks and one year was only small and not clinically relevant anymore because of ceiling effects in the “normal decrease of pain” and “no very little pain” groups for VAS and for all KOOS recovery trajectories (“gain group” and “moderate gain” group). Patients in the “sustained pain” group showed clinically relevant improvements between six weeks and one year. For the TUG classes there was also a clinically relevant improvement between six weeks and one year. Some studies showed most improvement in the first 3–4 months after surgery, followed by a ceiling effect after one year [15, 17, 18]. In our study the ceiling was nearly reached already at 6 weeks, while in patients with “sustained pain” a clinically relevant improvement took place later than in the other groups between six weeks and one year [40–43] which implies that these patients needed more time to recover than the first six weeks. Patients with “sustained pain” during the six weeks (*n* = 19), had the possibility to get pain free after one year (*n* = 15). At one year almost all patients were pain free, so the outcome was favorable for this group of patients.

The differences of the TUG and KOOS-ADL trajectories between the “gain” and “moderate gain” group was considered clinically relevant, while for VAS trajectories differences between “no/very low pain,” and “sustained pain” were statistically significant but small.

Our population included patients with a favourable prognosis and our data showed favourable recovery trajectories for most patients. If all patients after TKA surgery will be included in further research, we expect to find more or less similar recovery trajectories as in our population. Literature shows satisfaction rates of 80% after TKA, so in that case more patients will be in the “sustained pain” and “low gain” group.

### Further research

Recovery trajectories for patients after TKA including a high-intensity physiotherapy program were determined in the first weeks after TKA procedure. Studies looking at recovery trajectories in all patients after TKA, including those who followed other rehabilitation protocols and a less favorable prognosis are needed to generalize the results to all patients after TKA. Identifying patients at risk for less favorable outcomes (sustained pain or low physical function) is important to get realistic expectations of patients regarding the TKA surgery. This improves satisfaction after TKA [44]. Prognostic factors for outcome after TKA showed preoperative pain, preoperative physical function and anxiety as the best predictors for long-term outcome [20]. However as far as known this is not researched yet in studies looking at recovery trajectories, [20] so identifying preoperative prognostic factors for recovery trajectories is another recommendation for further research.

## Data Availability

The datasets used and analysed during the current study are available from the corresponding author on reasonable request.

## Appendix 1

**Table 3:** The high-intensity physiotherapy program

Aims program		Improving physical functioning Improving physical activity level in daily life Improving self-management skills
Inclusion criteria		For inclusion in the high-intensity physiotherapy program, the patient had to satisfy the following criteria: Patient 1) was undergoing primary TKA procedure for osteoarthritis 2) was physically able to complete the intensive program 3) had no comorbidity that hindered the patient in doing exercises two times a day 4) was independent in activities of daily living before surgery 5) had no psychological distress related to a higher risk of worse outcome (49,50) 6) had a BMI ≤ 30, (risk factor for worse outcomes) (51)) 7) provided informed consent
Hospital stay		Three days Physiotherapy was given two times a day with a mean duration of 18 min per session Physiotherapy consisted of education, isometric quadriceps muscle exercises, Range of Motion exercises, transfers, walking with a walking aid and walking stairs
Program	Location	Resort hotel
	Duration program	10 days
	Frequency PT	2 times a day
	Duration per PT session	1.5 h
	Intensity	High
	Content PT	Learning dynamic and symmetric walking pattern Range of Motion exercises (outside) walking on different surfaces cycling on a home trainer transfer training muscle strength exercises walking stairs aqua therapy fall prevention education
Discharge home		After the program patients were advised to exercise by themselves or continue physiotherapy in the home environment if necessary till goals were achieved in activities of daily living, sport and work activities.

## Appendix 2

**Table 4:** The BIC values for linear and quadratic models of trajectories of TUG, KOOS-ADL and VAS-pain for 0–6 weeks

Number of classes	TUG (linear model)	TUG (quadratic model)	KOOS-ADL (linear model)	KOOS-ADL (quadratic model)	VAS-pain (linear model)	VAS-pain (quadratic model)
2	2990.96	2988.98	4633.58	4637.66	5457.56	5453.77
3	3004.66	2959.98	4639.53	4643.57	5435.79	5432.35
4	2972.86	2969.78	4648.19	4642.88	5444.58	5440.46
5	2989.02	2985.63	465,408	4652.41	5453.33	5449.60

KOOS-ADL = Knee Osteoarthritis and Outcome Score- Activities of Daily Living; TUG = Timed Up and Go; VAS = Visual Analogue Scale
